# Neuropsychiatric and Cognitive Sequelae of COVID-19

**DOI:** 10.3389/fpsyg.2021.577529

**Published:** 2021-03-02

**Authors:** Sanjay Kumar, Alfred Veldhuis, Tina Malhotra

**Affiliations:** ^1^Department of Psychology, Oxford Brookes University, Oxford, United Kingdom; ^2^Oxford Health Foundation NHS Trust, Oxford, United Kingdom

**Keywords:** coronavirus disease 2019, neuropsychiatric disease, cognition, depression, mental health

## Abstract

Coronavirus disease 2019 (COVID-19) is likely to have long-term mental health effects on individuals who have recovered from COVID-19. Rightly, there is a global response for recognition and planning on how to deal with mental health problems for everyone impacted by the global pandemic. This does not just include COVID-19 patients but the general public and health care workers as well. There is also a need to understand the role of the virus itself in the pathophysiology of mental health disorders and longer-term mental health sequelae. Emerging evidence suggests that COVID-19 patients develop neurological symptoms such as headache, altered consciousness, and paraesthesia. Brain tissue oedema and partial neurodegeneration have also been observed in an autopsy. In addition, there are reports that the virus has the potential to cause nervous system damage. Together, these findings point to a possible role of the virus in the development of acute psychiatric symptoms and long-term neuropsychiatric sequelae of COVID-19. The brain pathologies associated with COVID-19 infection is likely to have a long-term impact on cognitive processes. Evidence from other viral respiratory infections, such as severe acute respiratory syndrome (SARS), suggests a potential development of psychiatric disorders, long-term neuropsychiatric disorders, and cognitive problems. In this paper, we will review and evaluate the available evidence of acute and possible long-term neuropsychiatric manifestations of COVID-19. We will discuss possible pathophysiological mechanisms and the implications this will have on preparing a long-term strategy to monitor and manage such patients.

## Introduction

Coronaviruses are single-stranded RNA viruses, which caused two well-known outbreaks: (1) severe acute respiratory syndrome (SARS) in 2002 and (2) Middle East respiratory syndromes (MERS) in 2012. Since December 2019, several cases of atypical pneumonia have been reported from Wuhan, China. A novel coronavirus was identified to be the cause and was subsequently named by the WHO as: “severe acute respiratory syndrome coronavirus 2 (SARS-CoV-2).” It is well known that coronaviruses affect the respiratory tract, with most patients experiencing only mild symptoms akin to the common cold (e.g., blocked/runny nose, headaches, sneezing, a raised temperature, loss of taste and smell, etc.) and the illness is self-limiting. However, if the virus reaches lower respiratory tract, in vulnerable individuals such as new-born, elderly, and immunocompromised, it can cause severe illness such as pneumonia, bronchitis, exacerbation of asthma, chronic obstructive pulmonary disease (COPD), and acute respiratory syndrome (ARS), as seen in SARS, MERS, and now COVID-19 (Desforges et al., 2014; Raj et al., 2014). Unfortunately, coronavirus is an opportunistic virus and can allude immune response, potentially spreading to cells other than the respiratory tract’s epithelial cells. Several coronaviruses have shown to be neuro-invasive, including SARS and MERS (Gu et al., 2005; Xu et al., 2005; Arabi et al., 2015).

As of the 28th of June 2020, SARS-CoV-2 has now infected over 10 million people worldwide (Dong et al., 2020) and the pandemic continues to grow. The disease caused by SARS-CoV-2 is known as corona virus disease 2019 (COVID-19), which manifests not just as a respiratory illness but also impacts the cardiovascular, renal, and the nervous system functions (Yuki et al., 2020). It is known from previous pandemics, such as Spanish influenza and SARS, that there are not just acute effects of the viral infection but also long-term sequelae due to disease itself as well as social effects due to governmental measures of containment such as quarantine, social distancing, and lockdown.

In this paper, we aim to present an understanding of pathophysiology, possible effects of SARS-CoV-2 infection to the brain and its long-term neuropsychiatric and cognitive consequences. Understanding neuropsychiatric and cognitive consequences are important, as millions of individuals have been affected; many more are undetected, and the number of infections is still rising. If even a fraction of such individuals experience neuropsychiatric complications, the public health implications could be considerable. Therefore, it is important to understand the neuropsychiatric and cognitive consequences of COVID-19. In this paper, we will briefly outline how COVID-19 can affect the central nervous system (CNS), review emerging evidence of effects on CNS and explore the possible neuropsychiatric sequelae of the COVID-19 infection. We will discuss diverse neuropsychiatric and cognitive complications following COVID-19 infection, possibly affecting a large proportion of individuals previously suffering from COVID-19. This, in turn, could lead to a potential increase in patients with psychiatric and cognitive problems. Understanding and assessing cognitive consequences following COVID-19 is important as it could be used to estimate an individual’s capacity to work effectively, drive, manage finances, participate in daily family activities, or make informed decisions. Appropriate neuropsychological rehabilitation could beplanned to remediate or compensate for cognitive deficits in COVID-19 survivors.

Neuropsychiatric consequences are neurological, psychiatric, and cognitive problems due to direct brain damage, disease, or indirect effects on the CNS *via* an immune response or medical therapy (Rogers et al., 2020). The acute psychiatric manifestations of COVID-19 reported in surveys are increased stress, anxiety, and depression (Asmundson and Taylor, 2020). In the long-term, psychiatric presentations could also be affected by the outcome of their illness, stigma or memories, and amnesia associated with the critical care they receive (Jones et al., 1998). Acute neurological symptoms such as headache, altered sensorium, acute cerebrovascular incidents, convulsions, and ataxia have been reported in more than a third of hospitalized patients (Mao et al., 2020). Reports of acute cognitive complications such as attention and dysexecutive symptoms are also emerging (Rogers et al., 2020; Varatharaj et al., 2020). However, we can just speculate about the long-term neuropsychiatric and cognitive consequences of COVID-19.

## Pathophysiology of Neuropsychiatric and Cognitive Consequences of Covid-19

SARS-CoV-2 is a novel virus and its pathophysiological mechanism on various physiological systems is yet to be fully understood. However, a lot can be learnt from other subtypes of coronaviruses. Coronaviruses primarily affect upper respiratory tracts, but they have been detected both in the brain and cerebrospinal fluids of the infected individuals (Bohmwald et al., 2018). There are several mechanisms through which coronaviruses can damage the nervous system. These may include direct infection injury, virus entering through blood circulation pathway, neuronal pathway, hypoxic injury, immune injury, and *via* binding to the angiotensin-converting enzyme 2 (ACE2). The neurotropic capacities of coronaviruses allow them to evade the immune response of the host and achieve latency. This makes them a potent factor to cause acute and late neurological effects. Although early indication shows that the expression of SARS-CoV-2 in the brain deviates slightly compared to SARS-CoV-1 and MERS expression, it is still a potential source for causing short and long-term neuropsychiatric and cognitive complications. For a detailed discussion of these mechanisms, please see Wu et al. (2020). The neuronal pathway *via* the olfactory nerve and role of ACE2 has been observed to be the primary pathophysiological mechanisms contributing to neuropsychiatric and cognitive complications in COVID-19 (Mirfazeli et al., 2020; Pantelis et al., 2020). This is mainly because coronaviruses affect the respiratory tract and can reach the ACE2-enzymes in the respiratory epithelial cells, and the olfactory nerve, providing a pathway for the coronavirus to enter the CNS.

### Neuronal Pathway

Neurotropic viruses, such as coronaviruses, use sensory and motor neuronal pathways to enter the CNS. One example of a neuronal pathway is the olfactory nerve (Desforges et al. 2019). This is mediated by the unique organization of olfactory nerves and the olfactory bulb in the nasal cavity and forebrain. The virus thus can reach the brain and CSF, which can cause inflammation and a demyelinating reaction. If the infection is set, then the viruses can reach the whole brain and CSF in less than 7 days (Bohmwald et al., 2018). Altered olfaction and gustatory problems (anosmia, hyposmia, and ageusia) have been reported in 49% of COVID-19 patients (Hornuss et al., 2020; Vaira et al., 2020) implicating the possibility of CNS infection through the olfactory neuronal pathway.

### ACE2 and Its Role in Neuropsychiatric Complications

ACE2 enzyme is widely present in various organs including oro-nasal, respiratory, cardiovascular, cerebrovascular, and immune systems. The high density of ACE2 in oro-nasal mucosa and their binding with SARS-CoV-2 may account for olfactory symptoms of anosmia in COVOD-19 (Lechien et al., 2020). Coronaviruses directly bind to ACE-2 receptors in respiratory epithelial cells cause cytokine storm, which causes widespread inflammation in patients with COVID-19, leading to multiple organ damage and immune-mediated encephalopathy manifesting as delirium and convulsions. Neuroinflammation is a well-recognized mechanism for the development of psychiatric disorders (Yuan et al., 2019). It can also cause hypercoagulable states causing ischaemic stroke besides other vascular events (Fotuhi et al., 2020). ACE2 plays an important role in controlling blood pressure but binding to SARS-CoV-2 can cause an increase in blood pressure, which can increase the propensity to cerebral hemorrhage. This may also explain the increase in mortality in patients with COVID-19 with comorbid metabolic conditions such as hypertension, high body mass index and diabetes (Fang et al., 2020). It has also been proposed that the spike protein of SARS-CoV-2 can bind to ACE2 receptors in capillaries, breaking the blood-brain barrier and allowing the virus to enter the brain directly (Wu et al., 2020). Neurons have a high density of ACE-2 and high binding to coronaviruses if they cross the blood-brain barrier. SARS-CoV-2 can lie latent in the neurons of patients who recover from acute effects of COVID-19, increasing the risk of long-term consequences by causing demyelination and neurodegeneration (Lippi et al., 2020).

## Neuropsychiatric and Cognitive Effects of Coronavirus Infection

### Acute Effects

In the short-term, 20–40% of COVID-19 cases may present with neuropsychiatric complications, such as cerebrovascular events, headache, dizziness, encephalopathies, anosmia, ageusia, and mood problems (Bo et al., 2020; Crunfli et al., 2020; Lu et al., 2020; Mao et al., 2020; Mirfazeli et al., 2020; Troyer et al., 2020; Varatharaj et al., 2020; Wu et al., 2020; Zhang et al., 2020), see Table 1. The acute effect of CoV infections on the CNS is manifested in viral encephalitis, infectious toxic encephalopathy, and acute cerebrovascular disease. In a recent meta-analytic review, Rogers et al. (2020) reported the neuropsychiatric short- and long-term consequences of SARS and MERS infection. They reported that during the acute illness, 27–41% of cases had neuropsychiatric symptoms such as confusion, depressed mood, anxiety, impaired memory, and insomnia. Steroid-induced mania and psychoses were also reported. The meta-analysis also looked at the available data related to COVID-19 infection and neuropsychiatric consequences and found that confusion and agitation were present in 65–69% of the intensive care unit patients. Importantly, at discharge, 33% of the patients with COVID-19 had dysexecutive syndromes. Similarly, in a United Kingdom wide surveillance study, 153 patients were reported to have neurological and neuropsychiatric complications following COVID-19 infection (Varatharaj et al., 2020). Twenty-one of these cases developed a new diagnosable psychiatric disorder. The other noteworthy observation was the effect of age on altered mental status and cerebrovascular incident. Thirty-seven patients had altered mental status and 49% of these patients were younger than 60 years. However, of 74 patients who presented with cerebrovascular incidents, 81% of the patients were aged over 60. In another report, Mao et al. (2020) reported neurologic manifestations of COVID-19. The study had 214 patients with mild to severe COVID-19 infection. Around 36.4% of the patients showed neurologic manifestations involving the CNS, peripheral nervous system (PNS) and skeletal muscles. These neurologic manifestations were more prevalent in patients with a severe infection and included signs of impaired consciousness and acute cerebrovascular diseases. Autopsy results have indicated degenerated neurons and an increased blood flow to some regions together with edematous brain tissue (Mao et al., 2020).

**Table 1:** Percentage of COVID-19 patients showing neuropsychiatric and cognitive effects.

	Reference	COVID-19 patients showing neuropsychiatric and cognitive effects							
		CNS^1^	PNS^2^	Affective disorders	Anxiety	Fatigue	PTSD	Impaired attention	Impaired memory
Short-term	Bo et al., 2020						96%		
	Crunfli et al., 2020			20%	28%			45%	28%
	Lu et al., 2020	25%	35%^a^	42%		27%			13%
	Mao et al., 2020	53%	19%						
	Mirfazeli et al., 2020	40%	36%^b^						
	Varatharaj et al., 2020	62%		17%					26%
	Zhang et al., 2020			29%					
Long-term	Hampshire et al., 2020							0.57SD^c^	
	Lu et al., 2020	10%	22%	17%		7%			28%
	Woo et al., 2020			11%		17%		44%	50%

1 Central nervous system (CNS) includes dizziness, headaches, mental state, ataxia, seizure, and acute cerebrovascular disease.

2 Peripheral nervous system (PNS) includes an impaired sense of smell, taste, vision, and nerve pain.

a A count of PNS symptoms occurring, it is possible a single patient had multiple symptoms.

b Average of reported PNS symptoms.

C Significant SD away from the healthy control group, indicating cognitive impairments for groups with different levels of medical assistance, the value here is the SD for patients requiring hospitalization with a ventilator.

Structural brain abnormalities have also been reported with SARS-CoV-1 and MERS infections. In an MRI study looking into the post-infectious neurological consequences of MERS in three patients with severe neurologic syndrome found hyperintensities in the white matter of the parietal lobes, temporal lobes, frontal lobes, basal ganglia, and corpus callosum (Arabi et al., 2015). The neurological manifestations shown by these patients included: altered mental status (ranging from confusion to coma) ataxia, and focal motor deficits. Similarly, a study investigating autopsies of six SARS patients found evidence of edema and scattered red degeneration of the neurons (likely the result of neuronal hypoxia or ischemia), after finding evidence of SARS genome sequences in the hypothalamus and cortex (Gu et al., 2005). Structural brain abnormalities have been reported in COVID-19 patients too. Montalvan et al. (2020) have reported cases with brain abnormalities present in the bilateral thalamic, medial temporal lobes, hippocampus, and insular regions.

As reported by Helms et al. (2020), there are some findings of encephalopathy and reduced blood flow in the frontotemporal brain region following a COVID-19 infection. The extent to which COVID-19 infection leads to neurological damages and neuronal symptoms is currently still unknown. However, several studies have found patients with neurological diseases ranging from encephalitis to strokes (Mao et al., 2020; Moriguchi et al., 2020). Encephalitis alone is linked to an increased risk of a range of long-term sequelae such as epilepsy, bipolar disorders, psychotic disorders, anxiety disorders, cognitive problems, and dementia (Granerod et al., 2017).

### Long-Term and Chronic Neuropsychiatric Sequelae

It is known that the neural and immune cells can host latent CoV which could contribute to delayed neurologic and neuropsychiatric complications (Desforges et al., 2019). However, long-term neuropsychiatric sequelae of COVID-19 are currently unknown. We can speculate long-term effects from our understanding of the mechanisms of the COVID-19 on the CNS and evidence from long-term neuropsychiatric effects of SARS-CoV-1 and MERS. Lam et al. (2009) reported that 55% of survivors of SARS-CoV-1 had post-traumatic stress disorder (PTSD). Furthermore, depression was in 39%, pain disorder in 36.4%, panic disorder in 32.5%, and obsessive-compulsive disorder in 15.6% of SARS-CoV-1 survivors. In their meta-analytic review, Rogers et al. (2020) also reported long-term neuropsychiatric consequences of SARS and MERS infections in 10–20% of the cases, such as depressed mood, insomnia, anxiety, irritability, memory impairment, and fatigue. However, it is important to understand that the neuropsychiatric manifestations, such as PTSD, depression, or anxiety, following COVID-19 infection could also be a psychological reaction to being infected, being in intensive care unit or experiencing stigma of contracting the infection.

If similar proportions of long-term neuropsychiatric complications emerge following COVID-19, then we can expect a crashing wave of neuropsychiatric sequelae (Troyer et al., 2020), which will have huge implication for management of the stretched healthcare resources in every country. Besides the neuropsychiatric sequelae, long-term implications will be observed with many neurological problems. For example, loss of smell is considered one of the hallmark symptoms of COVID-19 infection implying CNS involvement. This might have long-term implications for neuro infections and neurodegenerative diseases. Indeed, loss of olfaction is considered an early manifestation in Parkinson’s disease (Doty, 2012; Chase and Markopoulou, 2020). Therefore, the emergence of cognitive symptoms following COVID-19 may indicate an underlying neurodegenerative process. Furthermore, individuals with certain immunocompromised neurological conditions such as multiple sclerosis (MS) may show alterations in their non-motor symptoms following COVID-19, which may indicate an underlying neurodegenerative process. Higher risk of developing Parkinson’s disease and MS has been previously linked to SARS-CoV-1 infection (Fazzini et al., 1992; Murray et al., 1994). Indeed, long-term assessment of cognition will become a critical part of the care pathway for such individuals.

### Long-Term Cognitive Sequelae

From the emerging evidence and our understanding of the mechanism of CoV in the CNS, one can expect to have a range of cognitive consequences of COVID-19 infection. Attention and dysexecutive symptoms have commonly been reported with COVID-19 (Rogers et al., 2020; Varatharaj et al., 2020). Hypoperfusion in the frontotemporal region of the brain has also been reported (Helms et al., 2020) as well as structural brain abnormalities thalamic and temporal regions (Montalvan et al., 2020). Considering the demyelinating nature of the viral infection in the CNS, we can expect common cognitive problems that characterize demyelinating illnesses (such as MS). The Symbol Digit Modalities Test (SDMT), a test to assess the speed of information processing, is an exceptionally reliable and sensitive cognitive test for MS patients (Benedict et al., 2017). Similarly, a link between loss of smell in COVID-19 patients and the prodromal phase of Parkinson’s disease should be kept in mind while examining long-term cognitive consequences. A large amount of research has shown that executive functions are primarily affected in the prodromal phase of Parkinson’s disease along with prominent memory problems (Fengler et al., 2017). Taken together, the long-term cognitive examination of COVID-19 survivors should at least include tests assessing attention, executive functions, learning, and memory as well as the speed of information processing.

### Implications for Monitoring Long-Term Neuropsychiatric and Cognitive Sequelae

It is clear from the past outbreaks of SARS-CoV-1 and MERS and current reports of neurological and neuropsychiatric complications following COVID-19 that a large number of survivors will experience a range of neuropsychiatric and cognitive sequelae. These are likely to affect their mental, physical, and cognitive well-being. Which, in turn, will affect their emotional, occupational, and financial situations. Some of these patients may develop a full-blown neurological or psychiatric illness, or some might experience mild cognitive problems and that will increase their risk of developing dementia. Early indications show that the cognitive domains of executive functions, attention, and memory appear to be affected by COVID-19. Furthermore, there are potential increases in affective disorders, anxiety, fatigue, and PTSD (Hampshire et al., 2020; Lu et al., 2020; Woo et al., 2020), see Table 1. These symptoms can be due to pathoplastic change in brain physiology where the COVID-19 infection may modify brain functions after infection, which can lead to the development of brain vulnerabilities that may increase the probability to develop psychological distress. It is also possible that these neuropsychiatric symptoms and disorders are the psychological reactions of having contracted COVID-19 and undergoing associated medical interventions. This complex nature of neuropsychiatric presentations can be understood through a careful study of case history, accompanied by standardized neuropsychological assessments. This will help clarify if the neuropsychiatric and cognitive problems are a direct consequence of structural brain abnormalities or are a psychological reaction of the potential physical and the mental stress associated with recovering from COVID-19. Therefore, early detection and prevention of neuropsychiatric and cognitive problems should be the long-term aim of health services and governments across the world as this could present as a “**third wave**” of the pandemic.

## Conclusion

The short-term neuropsychiatric and cognitive complications following COVID-19 are varied and affect a large proportion of COVID-19 survivors. In the medium- and long-term period, there is going to be an influx of patients with psychiatric and cognitive problems who were otherwise healthy prior to COVID-19 infection. Increased neuropsychiatric manifestations could be observed in the form of an increase in cases of depression, anxiety, PTSD, and in certain cases severe mental illnesses. Cognitive sequelae are also likely to be varied and a detailed cognitive evaluation should be considered for such individuals to monitor the emergence of new neurological cases. Robust neuropsychiatric and cognitive monitoring will enable health care providers to plan adequate health care delivery and allocate resources adequately. Early intervention for emerging cognitive problems will be critical for independent functioning and improved quality of life for many COVID-19 survivors.

## Author Contributions

All authors participated in the literature search, manuscript preparation, and feedback on final manuscript. SK led the process and conceptualized the topic. All authors contributed to the article and approved the submitted version.

### Conflict of Interest

The authors declare that the research was conducted in the absence of any commercial or financial relationships that could be construed as a potential conflict of interest.
